# Exercise-Induced Acute Bilateral Upper-Arm Compartment Syndrome

**DOI:** 10.1155/2017/9454782

**Published:** 2017-09-20

**Authors:** Brian C. Traub, Mark K. Lane, Jeff A. Traub

**Affiliations:** ^1^Emory University School of Medicine, Atlanta, GA, USA; ^2^Department of Orthopaedics and Rehabilitation, Detroit Medical Center, Wayne State University School of Medicine, Orthopaedic Surgery Residency Program, Detroit, MI, USA; ^3^Department of Orthopaedics, Dekalb Medical Center, Decatur, GA, USA

## Abstract

We present a rare case of acute exercise-induced bilateral upper-arm compartment syndrome in a patient who, after a year-long hiatus from exercise, subjected his upper-extremities to the stress of over 100 pushups. The patient presented with severe pain of the bilateral biceps and triceps and complaints of dark urine. Decompressive fasciotomy was performed followed by an intensive care unit (ICU) stay for associated myoglobinuria secondary to rhabdomyolysis. The patient suffered no long-term sequelae as a result of his conditions and recovered full function of the bilateral upper-extremities. Albeit rare, acute exercise-induced compartment syndrome should be considered as a diagnosis following unaccustomed bouts of exercise.

## 1. Introduction

Acute compartment syndrome is a condition defined by increased compartmental pressure which causes a decrease in perfusion pressure, ultimately leading to tissue ischemia. The condition is a clinical emergency, requiring prompt diagnosis and intervention. While most cases of acute compartment syndrome occur secondary to traumatic fracture, up to 30% occur without evidence of fracture [1, 2]. Other relatively common causes of acute compartment syndrome include complications of surgery, constricting circumferential casts, and thermal injuries [3]. While acute compartment syndrome can occur in any compartment, it is rare in the upper-arm. Although few cases have been reported in the literature, making ascertainment of the true prevalence difficult, trauma remains the most commonly reported cause of acute compartment syndrome in the upper-arm [3]. Reports of bilateral acute upper-arm compartment syndrome are even fewer, with most cases attributable to those aforementioned etiologies. We present a rare case of acute exercise-induced bilateral upper-arm compartment syndrome.

## 2. Case Report

A 24-year-old man presented to the emergency department complaining of left shoulder pain. The patient reported completing over 100 pushups 48 hours prior to presentation, but denied pain immediately after the workout. Prior to those pushups, he had taken a year-long hiatus from exercise. Plain radiographs of the left shoulder were negative and the patient was discharged and instructed to follow-up with orthopedics.

The patient returned to the emergency department after 24 hours, complaining of increased swelling in both arms and darkening of his urine. Blood testing returned a normal complete blood count, normal sedimentation rate, negative urine toxicology screen, myoglobin of 176 ng/mL, and a chemistry panel with a Na of 133 mmol/L, Cl of 97 mmol/L, Ca of 9.1 mg/dL, K of 4 mmol/L, and creatine kinase of 215,420 U/L. Urinalysis was significant for 3+ blood and 1+ protein with fewer than 1 RBC and 1 WBC per high power field. The patient was diagnosed with rhabdomyolysis and admitted.

After further evaluation, given a normal sedimentation rate, the admitting service had no concern for occult infection but they noted upper-extremity swelling, concerning for compartment syndrome. Orthopedics was immediately consulted. On examination, the patient appeared to be in moderate to severe pain with very tense bilateral upper-extremities, especially about the triceps but also involving the biceps. Passive range of motion at the elbow exacerbated his pain involving the upper-arm compartments. Muscle strength was graded at 3/5 in both the triceps and biceps bilaterally. Radial and ulna pulses were 2+ bilaterally. Compartment pressures were obtained by injecting saline into the affected compartments using a Stryker Intra-Compartmental Pressure Monitor (Kalamazoo, MI) which returned readings of 36 mmHg and 72 mmHg in the left forearm and right triceps, respectively. Additional compartments were not tested as the decision to go to the operating room was made upon recording the aforementioned pressures. In light of the clinical presentation, an absolute compartment pressure of >30 mmHg was used as the cutoff for operative management. Compartment pressures of 0–8 mmHg were considered normal [4].

Compartment pressures and clinical findings were consistent with compartment syndrome and the patient underwent emergent bilateral upper-extremity fasciotomies utilizing a posterior and medial approach to the upper-arm extending from the axilla to the level of the humeral epicondyles. Intraoperative inspection of the biceps and triceps musculature revealed a dusky grayish color beneath the fascia. Once the fascia was incised and the muscle was released, a majority of the involved muscle regained a pink color and was responsive to electrosurgical stimulus. Postoperatively, the patient reported immediate pain reduction. He was placed in the ICU for 3 days of continued fluid resuscitation with kidney function monitoring before transfer to the general medical/surgical floor. He was discharged 3 days later, upon normalization of his kidney function.

## 3. Discussion

Acute compartment syndrome, while less common in the upper-arm, is a surgical emergency with significant sequelae if not diagnosed and treated promptly. The case we reported is noteworthy because of its atypical nature. While most cases of acute compartment syndrome can be attributed to causes including trauma, surgery, or the placement of a circumferential cast or bandage, it can reasonably be inferred that our case is the result of exercise intolerance. This notion is supported when considering our patient's clinical picture and history. After burdening his upper-extremity musculature with over 100 pushups, the patient presented with bilaterally tense upper-extremity compartments, most severe in the compartments of the triceps. This is consistent with our proposed etiology, as the triceps are most heavily involved in the work associated with performing pushups.

The development of acute compartment syndrome following strenuous exercise may be attributable to the development of rhabdomyolysis [5]. Numerous cases of rhabdomyolysis following bouts of exercise have been reported. Tran et al. published a case report of a 23-year-old woman who developed rhabdomyolysis after exercise [6]. Similar to our patient, the woman had no significant medical history and developed symptoms after low-intensity and high-repetition exercise. The pathological manifestations of rhabdomyolysis are secondary to increased intracellular calcium levels, initiating a cascade of intracellular processes that culminates in outcomes including mitochondrial dysfunction and the production of reactive oxygen species [7]. Dysfunction of muscle cells, induced by rhabdomyolysis, promotes the accumulation of extracellular fluid within the cells, causing local edema formation with a subsequent increase in intramuscular pressure. The elevated pressure impedes muscle perfusion and venous return, resulting in muscle ischemia. The ischemic muscle leads to increased capillary permeability, thereby promoting a cycle of worsening local edema. This process has been hypothesized as a possible cause of acute compartment syndrome in rhabdomyolysis [7].

Other cases have been reported that support the association between intense exercise and the development of rhabdomyolysis with progression to acute compartment syndrome. DeFilippis et al. reported a case of exercise-induced rhabdomyolysis complicated by acute kidney injury and bilateral acute compartment syndrome of the thighs following a spinning-class [8]. Aynardi and Jones described a case similar to that of our patient in which bilateral upper-arm compartment syndrome was diagnosed after a vigorous cross-training workout [5].

Regardless of the etiology or site of compartment syndrome development, the same principles of diagnosis and treatment can be applied. To prevent irreversible tissue necrosis, prompt diagnosis with subsequent emergent surgical decompression with fasciotomy is mandated [9]. Appropriate interpretation of compartment measurements remains controversial, as some experts utilize the difference between diastolic blood pressure and the compartment pressure to guide decision-making, as opposed to absolute compartment pressures alone [10]. Ultimately, all compartment measurements should be interpreted in light of the clinical setting. In our case, the diagnosis was delayed and our patient experienced myonecrosis with resulting myoglobinuria that required intensive care monitoring and resuscitation but did not progress to acute tubular necrosis. While the patient discussed in this case returned for a six-month follow-up with no resultant long-term deficits in muscle function, the potential for irreversible tissue damage increases with increased duration and severity of the elevated compartment pressures. Retrospectively, an orthopedic consultation during the patient's initial visit to the emergency department may have been warranted but the subtle signs and symptoms that were present at that time may not have been alarming to the emergency department staff. With only subtle signs and symptoms available to the emergency department staff on the patient's initial presentation, a high level of suspicion would have been required for inclusion of compartment syndrome in the differential diagnosis. The conventional thinking remains that compartment syndrome occurs secondary to prolonged pressure on an extremity or following trauma or surgery. We therefore conclude that acute compartment syndrome should be considered in patients presenting with any signs or symptoms consistent with the diagnosis in the setting of a recent bout of intensive exercise.
